# The prevalent status and genetic diversity of porcine reproductive and respiratory syndrome virus in China: a molecular epidemiological perspective

**DOI:** 10.1186/s12985-017-0910-6

**Published:** 2018-01-04

**Authors:** Zhenhua Guo, Xin-xin Chen, Rui Li, Songlin Qiao, Gaiping Zhang

**Affiliations:** 1 Key Laboratory of Animal Immunology of the Ministry of Agriculture, Henan Provincial Key Laboratory of Animal Immunology, Henan Academy of Agricultural Sciences, Zhengzhou, 450002 People’s Republic of China; 2 College of Animal Science and Veterinary Medicine, Henan Agricultural University, Zhengzhou, 450002 People’s Republic of China; 3 Jiangsu Co-innovation Center for Prevention and Control of Important Animal Infectious Diseases and Zoonoses, Yangzhou, 225009 People’s Republic of China

**Keywords:** Porcine reproductive and respiratory syndrome virus (PRRSV), Molecular epidemiology, PRRSV-1, PRRSV-2, Control strategies

## Abstract

Porcine reproductive and respiratory syndrome virus (PRRSV) has been epidemic more than 30 years in America and 20 years in China. It is still one of the most important causative agents to the worldwide swine industry. Here, we systematically analyzed the prevalence status of PRRSV in China by a molecular epidemiological perspective. Now both PRRSV-1 and PRRSV-2 are circulating and approximately more than 80% of pig farms are seropositive for PRRSV. For PRRSV-2, there are four lineages (lineage 1, lineage 3, lineage 5, lineage 8) circulating in the fields. Lineage 8 (CH-1a-like) and lineage 5 (BJ-4-like) appeared almost at the same time during 1995-1996. Notably, BJ-4 shares 99.6% and 99.8% identity with VR2332 and RespPRRS MLV, respectively. It means that lineage 5 is likely to be imported from America. Now highly pathogenic PRRSV (HP-PRRSV) which was considered to be evolved from local diversity of lineage 8 strains is predominant with different variants. Lineage 3 appeared in 2010 which is mainly sporadic in south of China. Lineage 1, also known as NADC30-like strains in China, has been prevalent since 2013 and leads to PRRS pandemic again. For PRRSV-1, although sporadic at present, more than 9 provinces/regions have been reported. All the circulating strains belong to subtype I. It should be paid more attention since there are no vaccines available. Our analysis would help to deeply understand the prevalent status of PRRSV in China and provide useful information for prevention and control of porcine reproductive and respiratory syndrome (PRRS).

## Background

Porcine reproductive and respiratory syndrome (PRRS) is one of the most important economically diseases to the swine industry worldwide. It is estimated that the total economic losses caused by PRRS is about $664 million annually in the America, an increase from the $560 million annual cost estimated in 2005 [1]. PRRS was firstly described in America in 1987 [2], followed in Europe and Asia (1990-1992) [3–6]. The causative agent, porcine reproductive and respiratory syndrome virus (PRRSV), was isolated in 1991 in the Netherlands and 1992 in the United States with originally isolated strains named Lelystad Virus (the European prototypic strain) and Swine Infertility and Respiratory Syndrome (SIRS) virus (also known as ATCC VR-2332, the North American prototypic strain), respectively [7, 8]. The retrospective studies showed that the conserved serum samples were PRRSV antibody positive as early as 1979 in Canada [9]. It suggested that PRRSV had already circulated in swine population before 1979.

PRRSV is an enveloped, single positive-stranded RNA virus. It belongs to the family *Arteriviridae* of the order *Nidovirales* along with equine arteritis virus, lactate dehydrogenase-elevating virus and simian hemorrhagic fever virus [10]. Its genome size is about 15.4 kb, which contains at least 10 open reading frames (ORFs) flanked by two untranslated regions 5′ and 3′ (5’UTR-ORF1a-ORF1b-ORF2a-ORF2b-ORF3-ORF4-ORF5/ORF5a-ORF6-ORF7-3’UTR). ORF1a and ORF1b encode two large polypeptides which produce 14 non-structural proteins by the viral enzymatic cleavage. ORF2 through ORF7 code eight structural proteins including GP2, E, GP3, GP4, GP5a, GP5, M and N [11–14]. Among them, ORF5 (gp5) is usually used for phylogenetic analyses because of its high variability. There are two genotypes of PRRSV: PRRSV-1 (European type) and PRRSV-2 (North American type) [15, 16]. Although these two types of PRRSV cause similar clinical disease to the infected pigs, they share only 55–70% nucleotide and 50–80% amino acid similarity in their various genes [17].

High genetic diversity is a significant characteristic of PPRSV. Mang Shi et al. constructed the global classification system of PRRSV based on the comprehensive analysis of the complete ORF5 gene sequence. According to the classification system, the PRRSV-1 was divided into three subtypes (subtype 1-3) and the PRRSV-2 was classified as 9 lineages with several sublineages of each lineage [18, 19]. The virulence and antigenicity are also different and variable due to the genetic diversity. There are several highly pathogenic PRRSV (HP-PRRSV) strains which cause severe reproductive and/or respiratory diseases and the emergence of new HP-PRRSV often leads to the widespread re-pandemic of PRRS. For example, although the vaccine was used in the late 1990s, the “abortion storm” swept through the America and an atypical PRRSV strain MN184 was isolated in 2001 [20, 21]. In Eastern Europe, a highly pathogenic PRRSV, Lena strain, was reported by Belarus in 2010 [22]. In China, the emergence of HP-PRRSV variants in 2006 led to atypical PRRS pandemics and 20% mortality in pigs [23]. Since 2013, PRRS became prevalent again in China caused by new PRRSV variants, NADC30-like strains, which are considered to be imported from North American and adapted in China [24, 25].

Here, we retrospectively and phylogenetically analyzed the prevalence and genetic diversity of PRRSV in China through a molecular epidemiological perspective. We hope to provide valuable information and new insights for the PRRSV epidemic trends and control strategies. Genome analyses were performed using the DNASTAR package. The unrooted phylogenetic tree was generated by the distance-based neighbor-joining method using MEGA 6.0 and the amino acid sequences were aligned by Clustal W method (MEGA 6.0) [26]. The recombination events were detected by RDP4 program using seven different algorithms (RDP, GeneConv, BootScan, MaxChi, Chimera, SiScanm and 3Seq) with Bonferroni correction and a highest acceptable *p*-value of 0.01 [27].

## PRRSV history and prevalent status in China

PRRS was firstly described in 1995 in China and the etiological agent, PRRSV, was isolated by Guo et al. (1996) and Yang et al. (1997). The isolated strains were named CH-1a (Genbank ID: AY032626) and BJ-4 (Genbank ID: AF331831), respectively. Both of them belong to PRRSV-2 [28, 29]. Serological studies showed that the percent of positive serum is more than 40% in the east and north of China [30], and it means that PRRSV circulated in China earlier than 1995. It is not clear how the PRRSV was introduced into pig population in China. One possibility is the import of breeding pigs. The evidences are: (i) Chen et al. (1996) successfully isolated PRRSV strain from the imported breeding pigs from Canada [31]; (ii) The genome homology of BJ-4 with VR2332 is about 99.6% and the genome homology of CH-1a with JA142 (Genbank ID: AY424271) is about 94.5%. Both of them (especially BJ-4) are closely related to North American isolates and PRRSV was endemic during 1995~2005.

During the summer of 2006, a new PRRSV variant, also known as highly pathogenic PRRSV (HP-PRRSV), caused atypical PRRS and led to a devastating destruction to swine industry with 20% motality to pigs. The new PRRSV variants show a unique discontinuous 30 amino acids (482aa, 534-562aa) deletion in Nsp2 gene [22]. The representative virus strains are JXA1 (Genbank ID: EF112445), TJ (Genbank ID: EU860248) and HuN4 (Genbank ID: EF635006) which are also the parent strains for attenuated vaccine strains JXA1-R, TJM-F92 and HuN4-F112, respectively [32–34]. The HP-PRRSV-like strains are predominantly epidemic strains in the pig farms after 2006. Since 2013, there was a markedly increased PRRSV infection during routinely clinical investigation. The reason for this re-pandemic of PRRS is considered as the emergence of NADC30-like strains which were probably imported from North America and went though extensive variation in China [24, 25, 35]. Now both HP-PRRSV-like and NADC30-like strains are mainly circulating in the fields and have a high clinical detection rate.

For the PRRSV-1, B13 (Genbank ID: AY633973) was the earliest strain reported in China. It was conserved in Dalian Animal and Plant Quarantine Bureau, whose original source is not clear [36]. The retrospective studies showed that the PRRSV-1 strains could be detected from clinical samples as early as 2006 in mainland of China [37]. And now they are sporadic in more than nine provinces/regions (Inner Mongolia, Heilongjiang, Liao-ning, Beijing, Fujian, Guizhou, Guangdong, Shanghai, Hong Kong) [38]. We should pay more attention to the prevalence of European type in China since more and more clinical investigation reported their existence and there are no authorised vaccines yet.

## The epidemic of PRRSV-2 in China

**Fig. 1:**
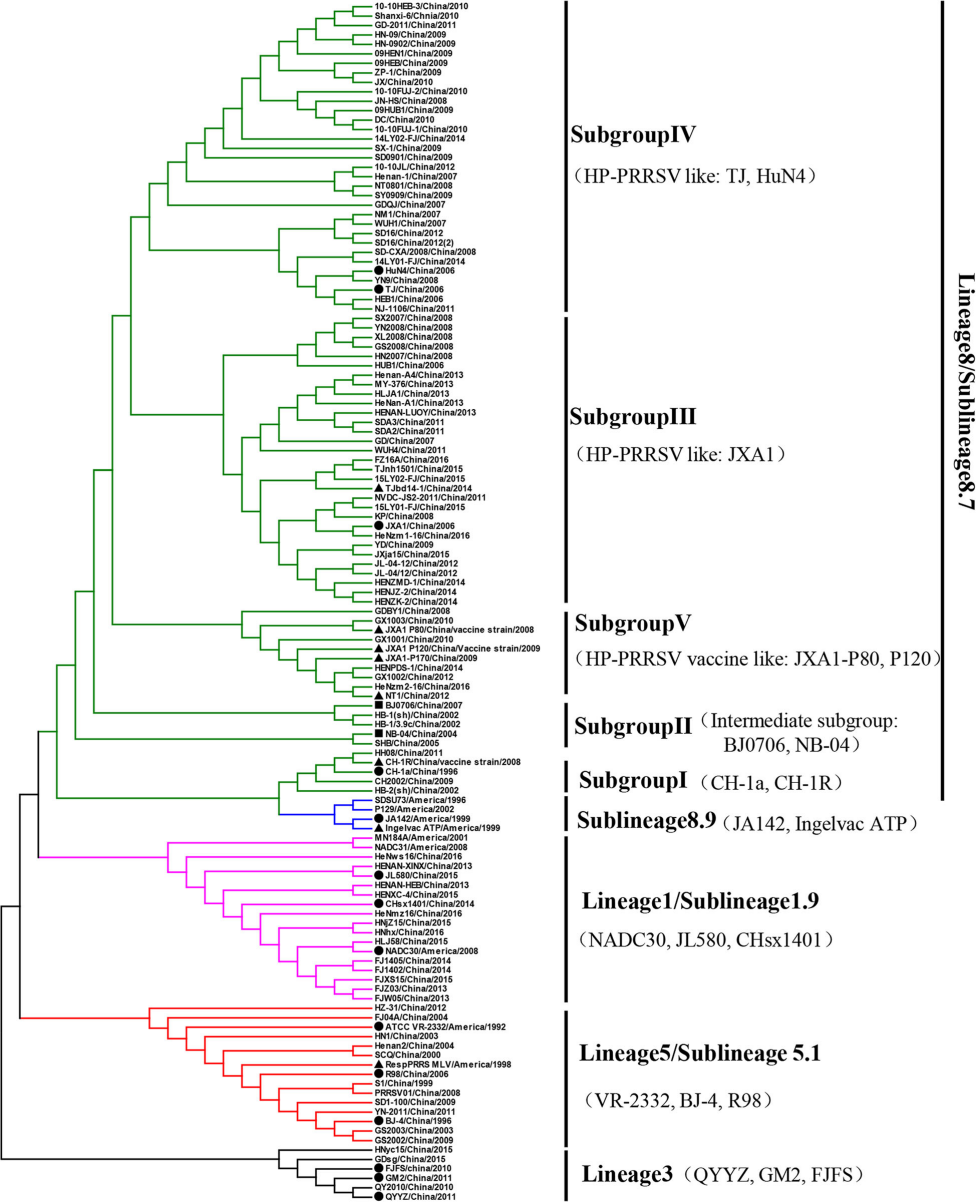
The phylogenetic analysis based on ORF5 genes from 127 reference virus strains of PRRSV-2. The unrooted phylogenetic tree was constructed using the distanced-based neighbor-joining method in MEGA6.0 with bootstrap values (1000 replicates). The PRRSV-2 circulating in China were clustered into four lineages. Lineage 8 is predominant in 1996-2016 in China and could be further subdivided into five subgroups. Lineage 1 spreads rapidly since 2013 in China. The lineages are according to previous report. The circles (●) indicate the representative virus strains of each lineage in China. The triangles (▲) mean the attenuated live vaccine strains or vaccine derivatives. The squares (■) represent two transition virus strains

Based on the global PRRSV classification system and ORF5 sequence information in the Genbank, we chose 127 PRRSV-2 ORF5 sequences, including 9 reference strains and 118 prevalent strains (Table 1). As shown in Fig. 1, the prevalent PRRSV-2 strains in China were clustered into four lineages: lineage 1, lineage 3, lineage 5 (sublineage 5.1) and lineage 8 (sublineage 8.7). Lineage 8 is predominant since the emergence of PRRSV in China which included classical PRRSV strains (CH-1a-like) prevalent before 2006 and HP-PRRSV-like strains prevalent after 2006. The epidemic of lineage 1, also known as NADC30-like strains, which spread rapidly around the country since 2013 and now the clinical detection rate is comparable with lineage 8 (HP-PRRSV-like). Lineage 3 was another newly emerged variants since 2010 which is mainly circulating in south of China (Jiangxi, Fujian, Guangdong, Guangxi) and the clinical detection rate is less than 10% [39, 40]. Although the lineage 5 (BJ-4-like/VR2332-like) appeared as early as in 1996, it is always non-pandemic in China and the clinical detection rate is low. It is still a puzzle that how the lineage 8 (HP-PRRSV-like) and lineage 1 (NADC30-like) become the major epidemic strains, while lineage 8 (CH-1a-like) and lineage 5 are always endemic in China. One reasonable explain is that HP-PRRSV-like and NADC30-like strains show high genetic variations and incidence of recombination, compared with lineage 8 (CH-1a-like) and lineage 5. These characteristics probably made current vaccines ineffective and confer them much easier to escape the immune surveillance. Thus, they adapted well during the pig populations.

**Table 1:** The reference sequence information of PRRSV-2

No.	Virus strain	Origin	Accession no.	No.	Virus strain	Origin	Accession no.
1	ATCC VR2332	USA, 1992	U87392	40	HN2007	China, 2008	EU880437
2	RespPRRS MLV	USA, 1994	AF066183	41	SX2007	China, 2008	EU880434
3	SDSU73	USA, 1996	JN654458	42	SD-CXA/2008	China, 2008	GQ359108
4	JA142	USA, 1997	AY424271	43	XL2008	China, 2008	EU880436
5	Ingelvac ATP	USA, 1999	DQ988080	44	YN2008	China, 2008	EU880435
6	MN184A	USA, 2001	DQ176019	45	GS2008	China, 2008	EU880431
7	P129	USA, 2002	AF494042	46	PRRSV01	China, 2008	FJ175687
8	NADC30	USA, 2008	JN654459	47	KP	China, 2008	GU232735
9	NADC31	USA, 2008	JN660150	48	YN9	China, 2008	GU232738
10	CH-1a	China, 1996	AY032626	49	JN-HS	China, 2008	HM016158
11	CH-1R	China, 2008	EU807840	50	GDBY1	China, 2008	GQ374442
12	BJ-4	China, 1996	AF331831	51	ZP-1	China, 2009	HM016159
13	S1	China, 1999	DQ459471	52	SD1-100	China, 2009	GQ914997
14	SCQ	China, 2000	DQ379479	53	GS2002	China, 2009	EU880441
15	HB-1(sh)/2002	China, 2001	AY150312	54	CH2002	China, 2009	EU880438
16	HB-2(sh)/2002	China, 2001	AY262352	55	YD	China, 2009	JF748717
17	HB-1/3.9c	China, 2002	HQ233605	56	SX-1	China, 2009	GQ857656
18	GS2003	China, 2003	EU880442	57	SY0909	China, 2009	HQ315837
19	HN1	China, 2003	AY457635	58	09HEB	China, 2009	JF268679
20	Henan2	China, 2004	AY613349	59	09HEN1	China, 2009	JF268684
21	NB-04	China, 2004	FJ536165	60	09HUB1	China, 2009	JF268682
22	FJ04A	China, 2004	DQ246451	61	HN-0902	China, 2009	JX162590
23	SHB	China, 2005	EU864232	62	HN-09	China, 2009	JX174280
24	JXA1	China, 2006	EF112445	63	SD0901	China, 2009	JN256115
25	JXA1-p80	China, 2008	FJ548853	64	DC	China, 2010	JF748718
26	JXA1-P120	China, 2009	KC422727	65	GX1003	China, 2010	JX912249
27	JXA1-P170	China, 2009	JQ804986	66	QY2010	China, 2010	JQ743666
28	HuN4	China, 2006	EF635006	67	10-10HEB-3	China, 2010	JQ663553
29	TJ	China, 2006	EU860248	68	Shanxi-6	China, 2010	KJ855518
30	HEB1	China, 2006	EF112447	69	GX1001	China, 2010	JQ955657
31	R98	China, 2006	DQ355796	70	10-FUJ-2	China, 2010	JQ663547
32	HUB1	China, 2006	EF075945	71	10-FUJ-1	China, 2010	JQ663546
33	BJ0706	China, 2007	GQ351601	72	JX	China, 2010	JX317649
34	GD	China, 2007	EU825724	73	GM2	China, 2011	JN662424
35	WUH1	China, 2007	EU187484	74	QYYZ	China, 2011	JQ308798
36	Henan-1	China, 2007	EU200962	75	GD-2011	China, 2011	KC527830
37	NM1	China, 2007	EU860249	76	NJ-1106	China, 2011	JX880029
38	GDQJ	China, 2007	GQ374441	77	NVDC-JS2-2011	China, 2011	JQ715698
39	NT0801	China, 2008	HQ315836	78	HH08	China, 2011	JX679179
79	SDA3	China, 2011	JX878380	104	CHsx1401	China, 2014	KP861625
80	SDA2	China, 2011	JX878379	105	14LY01-FJ	China, 2014	KP780881
81	WUH4	China, 2011	JQ326271	106	14LY02-FJ	China, 2014	KP780882
82	YN-2011	China, 2011	JX857698	107	TJbd14-1	China, 2014	KP742986
83	HZ-31	China, 2012	KC445138	108	HENZMD-1	China, 2014	KT424216
84	GX1002	China, 2012	JQ955658	109	HENJZ-2	China, 2014	KT424217
85	SD16	China, 2012	JX087437	110	HENZK-2	China, 2014	KT424218
86	10-10JL	China, 2012	JQ663554	111	GDsg	China, 2015	KX621003
87	JL-04/12	China, 2012	JX177644	112	FJXS15	China, 2015	KX758250
88	SD16	China, 2012	JX087437	113	TJnh1501	China, 2015	KX510269
89	FJFS	China, 2012	KP998476	114	HNjZ15	China, 2015	KT945017
90	NT1	China, 2012	KP179402	115	HNyc15	China, 2015	KT945018
91	MY-376	China, 2013	KJ609517	116	JL580	China, 2015	KR706343
92	Henan-A4	China, 2013	KJ534539	117	HLJ58	China, 2015	KR706344
93	HeNan-A1	China, 2013	KJ002451	118	HENXC-4	China, 2015	KU950371
94	HENAN-HEB	China, 2013	KF416334	119	JXja15	China, 2015	KR149645
95	HENAN-XINX	China, 2013	KF611905	120	15LY01-FJ	China, 2015	KU215416
96	HLJA1	China, 2013	KT351739	121	15LY02-FJ	China, 2015	KU215417
97	HENAN-LUOY	China, 2013	KF416330	122	FZ16A	China, 2016	KY761966
98	FJZ03	China, 2013	KP860909	123	HeNhx	China, 2016	KX766379
99	FJW05	China, 2013	KP860911	124	HeNws16	China, 2016	MF474322
100	FJ1402	China, 2014	KX169191	125	HeNzm1-16	China, 2016	MF474323
101	HENJZ-2	China, 2014	KT424217	126	HeNzm2-16	China, 2016	MF474324
102	HENPDS-1	China, 2014	KT424228	127	HeNmz16	China, 2016	MF474321
103	FJ1405	China, 2014	KM453701

### Lineage 8/sublineage 8.7

According to the phylogenetic assay of ORF5 sequence, sublineage 8.7 could be divided into five subgroups—subgroup I~subgroup V (Fig. 1). Subgroup I is PRRSV strains that are closely related with CH-1a. CH-1a was the first isolated PRRSV strain in China and was recognized as an ancestor strain of classical PRRSV. The genome homology is 94.5% with JA142 (Genbank ID: AY424271) and SDSU73 (Genbank ID: JN654458) (sublineage 8.9) and 91.5% with VR2332 (sublineage 5.1). Subgroup II was considered as intermediate subgroup between classical and HP-PRRSV. BJ0706 (Genbank ID: GQ351601) and NB-04 (Genbank ID: FJ536165) were the representative strains of transition [41]. They have been found 1aa deletion at the 481th residue site in Nsp2 gene and they showed a genome identity of 95.1~95.8% with CH-1a and 97.1~98% with JXA1. Subgroup III~V belong to HP-PRRSV derivative strains or HP-PRRSV vaccine derivative strains. HP-PRRSV leaded to the outbreak of PRRS in China in 2006. The representative strains JXA1, TJ, HuN4 show 95.3% of genome homology with CH-1a and 89.6% with BJ-4. At present more consistent view is that HP-PRRSV is originated from lineage8 strains that have been circulating in China for about 10 years before the outbreak in 2006 [19]. The genetic diversity is still expanding and shows different evolutionary clusters following circulation of HP-PRRSV and extensive vaccination of modified live vaccines (JXA1-R, TJM-F92, HuN4-F112). Subgroup III is genetically closer with JXA1, also called JXA1-like group. Subgroup IV is evolutionarily close to TJ and HuN4 strains. Subgroup V is closely related with HP-PRRSV attenuated live vaccines. Considering modified live vaccines could circulate in the fields, it is expected to see many vaccines related sequences. And this also pose a challenge for a rapidly accurate diagnosis and control of PRRS.

### Lineage 5/sublineage 5.1 and lineage 3

Sublineage 5.1 appeared very early in China. The representative strain BJ-4 was isolated by Yang et al. in 1997 [29]. It shows 99.6% and 99.8% of genome homology with VR2332 and RespPRRS MLV (Boehringer Ingelheim, Germany), respectively. It suggests that sublineage 5.1 is more likely to be imported into China from North American. It is still not known why the prevalence of sublineage 5.1 is limited compared with lineage 8. Lineage 3 was firstly reported in 2010 and considered as a novel lineage originally [42, 43]. It is mainly endemic in southeast of China (Jiangxi, Fujian, Guangdong, Guangxi) [38]. The representative strains GM2 (Genbank ID: JN662424) and QYYZ (Genbank ID: JQ308798) show 86.2~88.7% of genome homology with VR2332, CH-1a, BJ-4 and JXA1. Lineage 3 is genetically distant from previous epidemic strains and its source remains to be further clarified. Clinical detection rate of both sublineage 5.1 and lineage 3 is less than 10%.

### Lineage 1

Lineage 1, also known as NADC30-like strains, has been reported since 2013 in China [44]. Now it’s epidemic in more than nine provinces/regions including northeast, southeast, central and east of China. The clinical detection rate is increasing every year and up to 50% in Henan Province [45]. The representative virus strain HENAN-XINX (Genbank ID: KF611905) and JL580 (Genbank ID: KR706343) share 86.7~87.8% genome homology with MN184A (Genbank ID: DQ176019) isolated in 2001 in America, 92.8~95.4% with NADC30 isolated in 2008 in America and 82.2~87.2% with CH-1a, BJ-4, JXA1 and QYYZ. Like MN184A and NADC30, the NADC30-like virus strain in China shows the identical deletion pattern of nsp2 including 131aa discontinuous deletion. NADC30 is likely to be introduced into China by import of breeding pigs [24, 25, 35].

Compared with the other PRRSV lineages in China, NADC30-like strains show more recombination possibilities and pathogenic diversity (Table 2). There are several reports about the recombination of NADC30-like strains including NADC30 with HP-PRRSV [24, 46–48], NADC30 with vaccine strain (TJM-like, JXA1-P80) [49–51] and with classical PRRSV (CH-1a, VR2332) [48, 52]. Besides, lineage 8 and lineage 3 also display different degrees of recombination [53, 54]. For the virulence difference, some NADC30-like strains (JL580, FJ1402 etc.) show high pathogenicity comparable with HP-PRRSV [24, 47, 51]. Some NADC30-like strains (TJnh1501, HNjz15 etc.) display intermediate virulence similar to the prototype strain-NADC30 [49, 55]. A reasonable explanation is that NADC30 underwent extensive mutations and recombination since it was introduced into China. Especially the exchange of gene fragment from different circulating strains which endow new biological characteristics to different variants, for example, the change of pathogenicity and antigenicity.

## The epidemic of PRRSV-1 in China

**Fig. 2:**
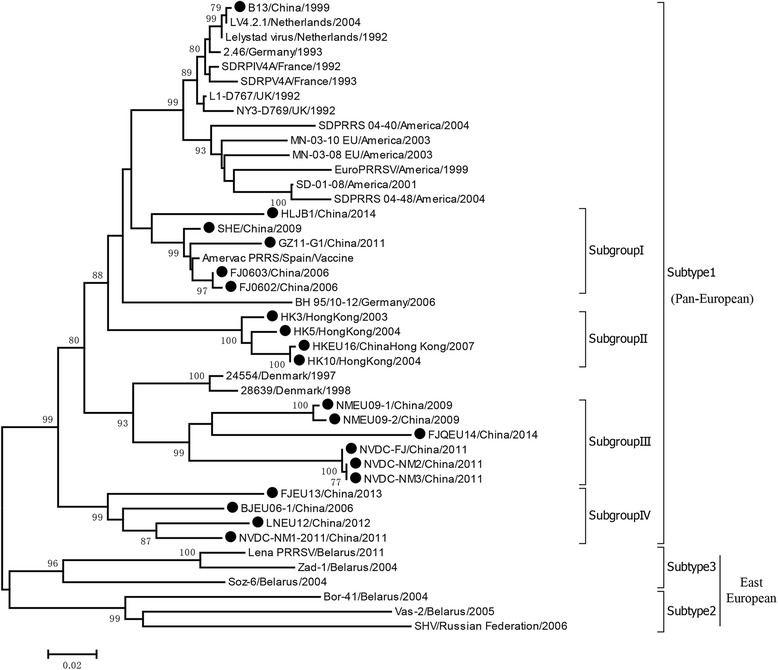
The phylogenetic assay based on ORF5 gene sequences of PRRSV-1. All the Chinese isolated strains belong to subtype 1 (pan-European) and can be classified into 4 subgroups according to the phylogenetic relationships. The Chinese PRRSV strains are marked with black circles (●)

Although there are more recent reports about the prevalence of European type PRRSV in China, the clinical detection rate is still low [37, 56–58]. Now the research mainly focuses on the molecular epidemiology of PRSSV-1 in China. Here we did a genetic diversity assay based on the ORF5 sequence by choosing 20 strains isolated in China and 23 reference strains (Table 3). The results showed all the Chinese isolated strains belong to subtype 1 (pan-European type) and could be further divided into four subgroups (Fig. 2). Our analysis may provide valuble information for the classification of future PRRSV-1 strains for other researchers.

**Table 2:** The recombinant virus strains and the recombination regions

No.	Recombinant strains	Major parent	Minor parent	Recombination regions	Virulence	Reference
1	JL580^a^	NADC30^a^	HP-PRRSV (09HEN1)	Nsp2, Nsp3~Nsp7, ORF2a~ORF4	Highly Pathogenic	[23]
2	FJ1402^a^	NADC30^a^	HP-PRRSV (GD)	Nsp2~Nsp3, Nsp12~ORF3	Highly Pathogenic	[47]
3	HENAN-HEB^a^	15HEN1^a^	HP-PRRSV (JXA1)	Nsp2		[48]
4	TJnh1501^a^	CHsx1401^a^	TJbd14-1^b^	Nsp2	intermediate virulence	[49]
5	FJW05^a^	JXA1-P80^b^	FJZ03^a^	Nsp1-ORF5	Highly Pathogenic	[50]
6	FJXS15^a^					[51]
7	HNhx^a^	HENAN-HEB^a^	HP-PRRSV (JXA1)	Nsp4~Nsp9		[46]
8	HNyc15^a^	15SC3^a^	CH-1a/VR2332	ORF2~ORF4		[52]
9	HENAN-XINX^a^	NADC30^a^	VR-2332	Nsp2~Nsp5		[48]
10	Chsx1401^a^	HENAN-XINX^a^	VR-2332	Nsp11	intermediate virulence	[49]
11	SY0909	HP-PRRSV (JXA1)	NT0801	Nsp12~ORF5		This study
12	GDsg	QYYZ	JXA1-P80^b^	Nsp9~Nsp11		[82]
13	GM2	QYYZ	RespPRRS MLV^b^	Nsp7~Nsp11		[54]
14	HB-1(sh)/2002	HB-1/3.9c	CH-1a	ORF2a~ORF4		This study
15	HNjz15	/	/	/	intermediate virulence	[55]

“^a^” means NADC30 or NADC30-like virus strains. “^b^” indicates modified live vaccine strains or vaccine derivative strain. The recombination events were detected by RDP4 program using seven different algorithms (RDP, GeneConv, BootScan, MaxChi, Chimera, SiScanm and 3Seq)

**Table 3:** The reference sequence information of PRRSV-1

No.	Virus strain	Origin	Accession no.	Subtype	No.	Virus strain	Origin	Accession no.	Subtype
1	Lelystad	Netherlands, 1991	M96262	Subtype1	23	SDRPIV4A	France, 1992	AY035919	Subtype1
2	BJEU06-1	Beijing, China, 2006	GU047344	Subtype1	24	NY3-D769	England, 1992	AY035940	Subtype1
3	NMEU09-1	Inner Mongolia, China, 2009	GU047345	Subtype1	25	L1-D767	England, 1992	AY035939	Subtype1
4	NMEU09-2	Inner Mongolia, China, 2009	GU047340	Subtype1	26	24,554	Denmark, 1997	AY035910	Subtype1
5	SHE	Shanghai, China, 2009	GQ461593	Subtype1	27	28,639	Denmark, 1998	AY035912	Subtype1
6	NVDC-NM1	Inner Mongolia, China, 2011	JX187609	Subtype1	28	LV4.2.1	Netherlands, 2004	AY588319	Subtype1
7	NVDC-NM2	Inner Mongolia, China, 2011	KC492504	Subtype1	29	EuroPRRSV	USA, 1999	AY366525	Subtype1
8	NVDC-NM3	Inner Mongolia, China, 2011	KC492505	Subtype1	30	SD-01-08	USA, 2001	DQ489311	Subtype1
9	NVDC-FJ	Fujian, China, 2011	KC492506	Subtype1	31	MN-03-08_EU	USA, 2003	AY749385	Subtype1
10	FJEU13	Fujian, China, 2013	KP860912	Subtype1	32	MN-03-10_EU	USA, 2003	AY749389	Subtype1
11	FJQEU14	Fujian, China, 2014	KP860913	Subtype1	33	SDPRRS 04-40	USA, 2004	EF175566	Subtype1
12	B13	China, 1999	AY633973	Subtype1	34	SDPRRS 04-48	USA, 2004	AY749411	Subtype1
13	FJ0603	Fujian, China, 2006	HM114313	Subtype1	35	Amervac	Spain,Vaccine	GU067771	Subtype1
14	GZ11-G1	Guizhou, China, 2011	KF001144	Subtype1	36	BH_95/10-12	Germany, 2006	JN651738	Subtype1
15	LNEU12	Liaoning, China, 2012	KM196101	Subtype1	37	2.46	Germany, 1993	AY035923	Subtype1
16	HKEU16	Hong Kong, China, 2007	EU076704	Subtype1	38	Vas	Belarus, 2005	DQ324689	Subtype2
17	HLJB1	Heilongjiang, China, 2014	KT224385	Subtype1	39	SHV	Russia, 2006	EU071236	Subtype2
18	HK3	Hong Kong, China, 2003	KF287129	Subtype1	40	Bor-41	Belarus, 2004	DQ324671	Subtype2
19	HK5	Hong Kong, China, 2004	KF287130	Subtype1	41	Lena	Belarus, 2007	JF802085	Subtype3
20	HK10	Hong Kong, China, 2004	KF287131	Subtype1	42	Zad-1	Belarus, 2004	DQ324694	Subtype3
21`	FJ0602	Fujian, China, 2006	HM755885	Subtype1	43	SOZ-6	Belarus, 2004	DQ324686	Subtype3
22	SDRPV4A	France, 1993	AY035920	Subtype1					

### Genetic variation

**Fig. 3:**
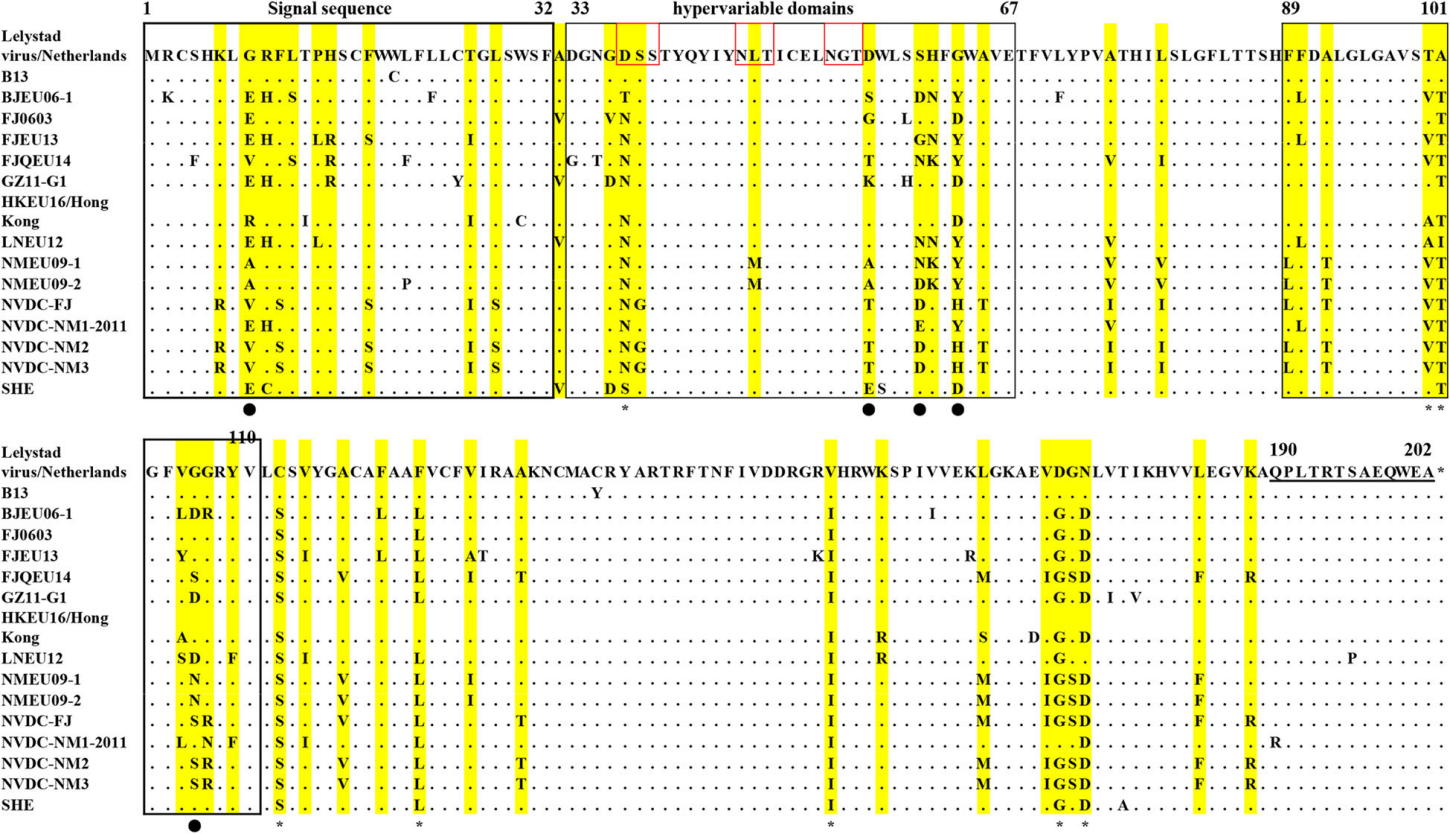
The amino acid alignment of ORF5 for PRRSV-1. The amino acid sequences were aligned by Clustal W method in MEGA 6.0. The signal sequence(1-32aa) and hypervariable domains (33-67aa, 89-110aa) were indicated by *solid boxes*. Potential N-glycosylation sites were shown in the *red boxes*. The hypervariable mutation sites were marked with dark spot (●) and the highly conserved mutation sites were indicated by asterisk (*)

The genome homology between Chinese isolated strains and Lelystad virus ranges from 85.9% to 92.7%, and the similarity of ORF5 with Lelystad virus is about 83.5~94.6% except the B13 which shows 99.7%, only two nucleotides different from Lelystad virus. It suggests that B13 may be isolated from import breeding pigs or as a standard reference strain from some western European countries. Considering the close communication of pig breeding between China and Europe, it is not surprising to see the appearance of PRRSV-1 in China. As the phylogenetic tree showed, The Chinese PRRSV-1 isolations are further clustered into four subgroups. Subgroup I was closely related with Amervac PRRSV virus, a live vaccine virus of PRRSV-1 and SHE, a rescued virus from an infectious clone of Amervac PRRS virus. Subgroup II was mainly limited in HongKong and it is geographically restrictive. Subgroup III is NMEU09-1/2-like isolates and subgroup IV is BJEU06-1-like strains. According to the amino acid alignment of ORF5 (Fig. 3), the 8/60/63/106 sites were highly variable which have 3 to 7 different amino acid mutations. The 37 (N/D^LV^), 100 (V/T^LV^), 101 (T/A^LV^), 112 (S/C^LV^), 123 (L/F^LV^), 155 (I/V^LV^), 173 (G/D^LV^) and 175 (D/N^LV^) are much conserved mutations in Chinese virus strains.

### N protein polymorphism

**Fig. 4:**
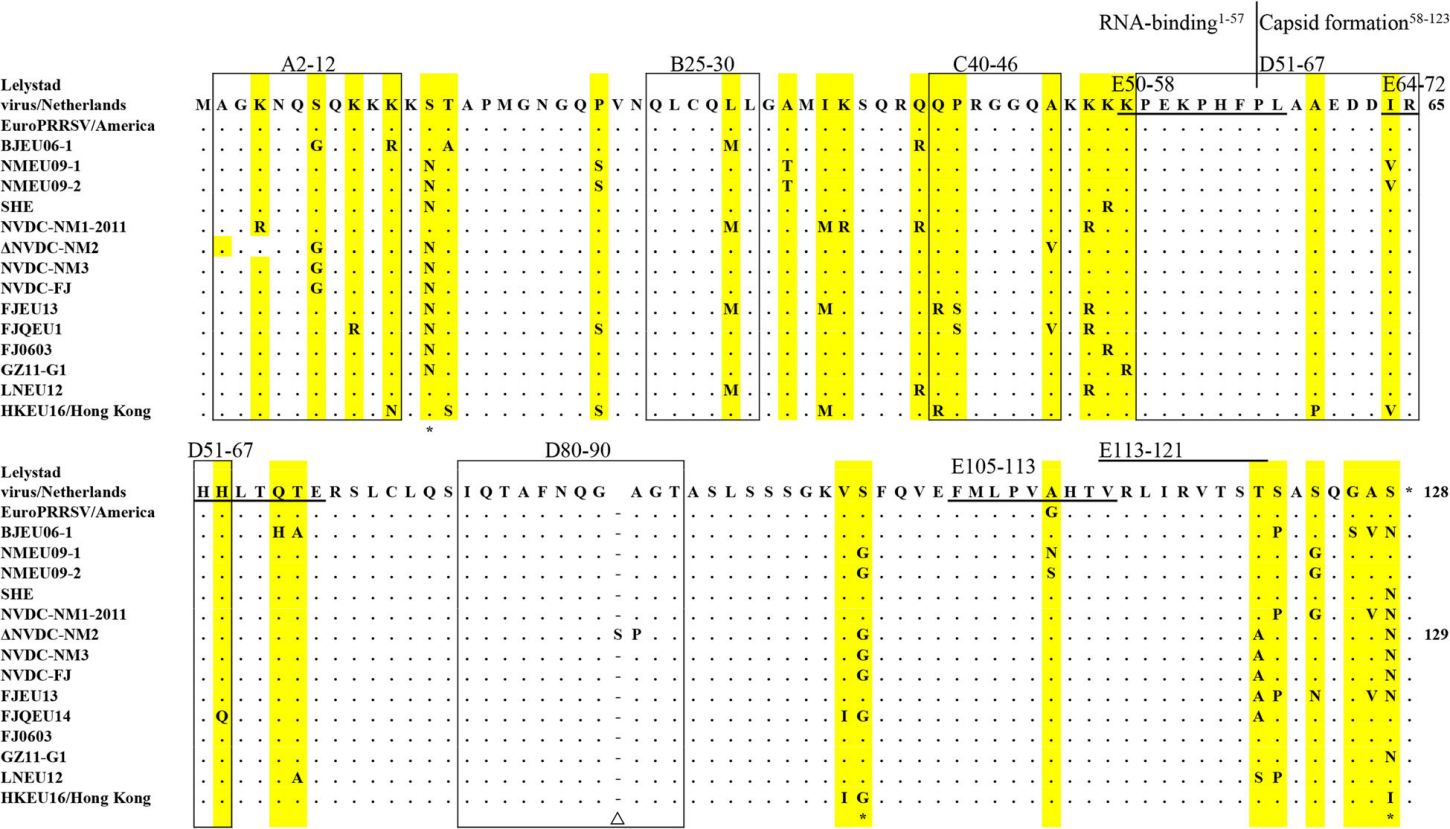
The alignment assay based on amino acid sequence of N protein for PRRSV-1. The sequence alignment was performed using Clustal W method. A2-12, B25-30, C40-46, D51-67 and D80-90 represent known epitopes in N protein; E50-58, E64-72, E105-113 and E113-121 indicate four porcine T –cell epitopes. The insertion site of amino acid was indicated by triangle (△). The highly conserved mutation sites in Chinese PRRSV-1 strains were marked with asterisk (*)

Normally the N protein is highly conserved. In genotype 1 PRRSV, N protein, as well as the high variable ORF5 and Nsp2 genes, is found to be very pleomorphic. The length of N protein ranges from 124 to 132aa [59–61]. Concretely, subtype 1 is 128aa (occasional 126/129/132aa), subtype 2 is 125aa (occasional 124/131aa), while subtype 3 is 124aa (occasional 128aa) [62]. In China, the N protein length of all the isolates is 128aa excepted NVDC-NM2 (Genbank ID: KC492504) strain which is 129aa and a Ser was inserted between 87th and 88th site. Based on the alignment assay (Fig. 4), the 13 (N/S^LV^), 100(G/S^LV^), 128(N/S^LV^) sites are conserved mutations among Chinese isolated virus strains.

### Pathogenicity and recombination

To our knowledge, until now only two Chinese PRRSV-1 isolations, GZ11-G1 and HLJB1, to be evaluated the pathigenicity. These two strains are pathogenic to piglets and can lead to classic PRRSV-specific lesions. The infectious pigs showed mild clinical signs and no pigs died, which indicated they are less virulent [58, 63]. Interestingly, both of them belong to subgroup I—the Amervac PRRSV vaccine-related strains. HLJB1 and GZ11-G1 show 91.64% and 96.3% genome homology with the Amervac PRRSV virus, respectively [58, 63]. Additionally, recombination analyses showed that HLJB1 is a recombinant from the Amervac vaccine and the BJEU06-1 isolate [64]. It is necessary to routinely monitor the prevalence of PRRSV-1 in the fields and more research needs bo be done for better understanding the biological and pathogenic characteristics of PRRSV-1 in China.

## Control strategies

### Vaccination

It is still a controversial question about the usage of PRRSV vaccines. On one hand, the vaccine efficiency is low or not significant from the clinical observation. On the other hand, there are no better choices currently under the circumstances of PRRSV pandemics. In China, there are more than seven commercial PRRSV vaccines (CH-1a/CH-1R, VR2332/Ingelvac PRRS MLV, R98/R98 MLV, JXA1/JXA1-R, TJ/TJM-F92, HuN4/HuN4-F112, GD/GDr180, etc) used. These vaccines are effective in reducing clinical signs, decreasing viremia and shortening duration of viral shedding. They can provide an efficient protection against a lethal challenge with their respective parental HP-PRRSV isolates [32–34, 65–67]. However, they can not completely prevent infection and establish sterilizing immunity. Also, the vaccine efficiency significantly decreases against heterologous challenge, and they can only provide partial or limited protection [47, 65, 68, 69]. The outbreak of HP-PRRSV in 2006 is in the backgroud of vaccination with CH-1a and Ingelvac PRRS MLV. Since 2013, NADC30-like virus strains transmitted quickly around herds in China although massive vaccination with all the commercial vaccines.

Massive vaccination with live attenuated vaccines also leads to safety concern and more genetic divergence: (i) It has been demonstrated that vaccine viruses could spread from vaccinated pigs to non-vaccinated, suggesting their ability to circulate in the fields and form different vaccine-associated clusters [70, 71]; (ii) The extensive immune pressure maybe serve as a major driving force which greatly promotes genetic diversity of genotype 2 in China [72]; (iii) Revertants from the vaccine derivatives should be paid more attention to. Indeed, there are several reports about the attenuated vaccine reverting to a virulent type [49, 73]; (iv) The frequency of recombination is increasing, especially the emergence of NADC30-like virus strains (lineage1) which display a wide broad recombination ability with HP-PRRSV (JXA1), classical PRRSV (CH-1a,VR2332) and attenuated vaccines (JXA1-P80, TJM-F92-like) [46, 49, 52, 74]. It has been verified that recombination is associated with re-prevalence of HP-PRRSV during 2009 to 2010 and closely related with the pandemic of NADC30-like virus since 2013 in China [24, 53]. Accordingly, veterinarians and farmers become more cautious and re-evaluate the previous immune strategies. Some farmers are trying to reduce and stop the vaccination of PRRSV in China by taking other comprehensive PRRS prevention and control measures.

### Production system and biosecurity

It is confirmed that multiple production sites and high quality biosecurity system are important in PRRSV prevention and control [75, 76]. Due to historical reasons, most of Chinese pig farms are one production site and the layout of gestation, farrowing, nursery and growing are also not reasonable. Many farms are still continuous flow sites and lack good biosecurity system. Following the development of swine production in China, more and more pig producers have recognized the significance of management, production system, environment control and good biosecurity. They have changed their views that only or over-dependent vaccination could prevent porcine diseases and would like to invest more in nutrition, management, environment, biosecurity and application of new technologies. For example, air-filtration ventilation systems were used by some breeding companines; gilts acclimation and strict batch production (also known as “All in All out”, AIAO) were accepted and implemented by more commercial pig farms.

### The strategy of “load, close, homogenise” (LCH)

The load, close, homogenise (LCH) strategy (also known as load, close, expose) was extensively accepted in PRRV control and elimination for it’s inexpensive and easy operability [75, 77, 78]. To perform this strategy, the producer should load enough gilts once for minimum of 200 days and then implement herd closure. The key points are gilts acclimation and good biosecurity system. Poul H. Rathkjen and Johannes Dall summarized ten golden rules for biosecurity management and showed a good model for successful implement of LCH [76]. Uniform PRRS status could be achieved either by simultaneous vaccination or by inoculation with serum containing resident virus. Notably, it should be very cautious when used the virus positive serum for acclimation because of the virulence of HP-PRRSV.

It is a better choice to build regional elimination of PRRSV. This model was successfully implemented in the state of Minnesota of the United States and in Horne Peninsula of the Denmark [75, 76]. They are good examples for HP-PRRSV regional elimination in China. This project needs close collaboration among veterinarians, pig producers and regional governments.

## Future perspective

Based on the ORF5 sequence analysis of PRRSV, we systematically retrospect the history and prevalence of PRRSV in China from a molecular perspective. PRRSV diverged earlier in the origin, and the early isolated strain CH-1a and BJ-4 only share 91.4% homology. CH-1a belongs to sublineage 8.7 and BJ-4 is clustered into sublineage 5.1. BJ-4 is likely to be imported from the North America or maybe as a vaccine strain since it shares 99.6% and 99.8% identity with VR2332 and RespPRRS MLV, respectively. It is still not clear why sublineage 5.1 is relatively stable and shows limited epidemicity. Nevertheless, sublineage8.7 is pandemic and goes through such genetic diversity, especially the emergence of HP-PRRSV which is devastating to swine production in China. Although the vaccines derived from HP-PRRSV play a role in reducing the severe clinical signs and slowing the prevalence of PRRS, it is still far away from expectation. And the massive usage of vaccines also lead to some side effects including virulence reversion of attenuated live vaccines [49, 73], markedly increase in virus recombination and variation [50, 53, 72, 74].

Recently, lineage 3 and lineage 1 are also circulating in Chinese swine herds. Lineage 3 is mainly limited in south of China (Jiangxi, Henan, Jiangsu, Guangdong, Guangxi) [38, 43]. Lineage 1 was firstly reported in Henan province in 2013 [45, 79], and then this lineage transmitted quickly in northeast, southeast, central and east of China. They lead to pandemic of PRRSV in China [38, 40, 80, 81]. The commercial vaccines seem not efficient since the dissemination of lineage 1 is under the background of massive vaccination. Several studies also show the commercial vaccines only provide limited or partial protection [38, 68, 69]. These facts prompt people to re-evaluate the strategies of prevention and control of PRRSV in China. And people become more cautious on massive vaccination. More and more farms try to improve management, biosecurity and environment instead of only over-dependent on vaccines.

Several studies showed the prevalence of PRRSV-1 in China, and up to 9 provinces and regions have been reported until now [38, 56–58]. All the Chinese circulating strains share some special common amino acids compared with Lylestad virus. Although the PRRSV-1 PRRSV is sporadic in China, we should pay more attention since there are no vaccines to be provided.

It has been 30 years since the first PRRS report in 1987 in America. Originally, we call it Mystery Swine Disease (MSD) [3, 7]. Thirty years later, although we have a deep understanding on clinical onset characteristics of PRRS, molecular epidemiology, virus proliferation, pathogenic mechanism, immune response and immune escape mechanism, PRRSV is still mysterious to us and we have not found a successful prevention and eradication strategy. There are still more questions need to be elucidated: i) What is the molecular mechanism of high variation and recombination frequency of PRRSV; ii) The broad spectrum neutralizing antibodies need to be analyzed further; iii) It remains to be elucidated that determination factors of virulence difference among different PRRSV strains; iv) How to look for a new vaccine development strategy which is safe, high efficient and have a broad spectrum protection to different PRRSV lineages. The clarification of these problems would provide great help and support for ultimate PRRSV control.

## Conclusion

PRRSV is still one of the most important causative agents to the swine production worldwide and causes huge economic losses every year. In China, although several commercial attenuated live vaccines have been widely used, PRRS is still severe in the pig industry. Moreover, the genetic diversity and complexity of PRRSV were further increasing. In this review, we systematically analyzed the PRRSV prevalent history and genetic evolution in China using a molecular epidemiological perspective. Additionally, we summarized the effective strategies and discussed the current problems in the prevention and control of PRRS. Our analysis would provide valuable information and new insights for the PRRSV epidemic trends and control strategies in China.

## Abbreviations

HP-PRRSV: Highly pathogenic PRRSV
ORFs: Open reading frames
PRRS: Porcine reproductive and respiratory syndrome
PRRSV: PRRS virus
SIRS: Swine infertility and respiratory syndrome
UTR: Untranlated region
